# Isoliquiritigenin Attenuates Monocrotaline-Induced Pulmonary Hypertension via Inhibition of the Inflammatory Response and PASMCs Proliferation

**DOI:** 10.1155/2019/4568198

**Published:** 2019-05-26

**Authors:** Haifeng Jin, Yang Jiang, Fengxia Du, Linna Guo, Guan Wang, Sang Chan Kim, Chul Won Lee, Lei Shen, Rongjie Zhao

**Affiliations:** ^1^Department of Anatomy, Qiqihar Medical University, Qiqihar, Heilongjiang 161006, China; ^2^Qiqihar Institute of Medical and Pharmaceutical Sciences, Qiqihar Medical University, Qiqihar, Heilongjiang 161006, China; ^3^Department of Etiology, Qiqihar Medical University, Qiqihar, Heilongjiang 161006, China; ^4^MRC-GHF, College of Korean Medicine, Daegu Haany University, Gyeongsan 38610, Republic of Korea; ^5^Department of Psychopharmacology, Qiqihar Medical University, Qiqihar, Heilongjiang 161006, China

## Abstract

Pulmonary hypertension (PH) is a progressive and serious disease, where exacerbated inflammatory response plays a critical role. Isoliquiritigenin (ISL), an important flavonoid isolated from Glycyrrhizae radix, exhibits a wide range of pharmacological actions including anti-inflammation. Previously we found ISL alleviated hypoxia-induced PH; in the present study, to extend this, we evaluated the effects of ISL on monocrotaline (MCT)-induced PH and the relevant mechanisms. Rats received a single intraperitoneal injection of MCT, followed by intragastric treatments with ISL (10 mg/kg/d or 30 mg/kg/d) once a day for 28 days. The MCT administration increased the right ventricular systolic pressure (RVSP) (*p* < 0.001), the median width of pulmonary arteries (*p* < 0.01), and the weight ratio of the right ventricular wall/left ventricular wall plus septum (Fulton index) (*p* < 0.01) in rats; however, these changes were inhibited by both doses of ISL (*p* < 0.05). In addition, treatment with ISL suppressed the upregulated production of serum interleukin-6 (*p* < 0.01) and tumor necrosis factor-*α* (*p* < 0.05) by MCT and reversed the increases in the numbers of proliferating cell nuclear antigen (PCNA)-positive cells (*p* < 0.01) in the medial wall of pulmonary arteries. In in vitro experiments, ISL (10 *μ*M, 30 *μ*M, and 100 *μ*M) inhibited excessive proliferation of cultured primary pulmonary artery smooth muscle cells (PASMCs) (*p* < 0.05,* p* < 0.01, and* p* < 0.001) in a dose-dependent manner and prevented an increase in the expressions of PCNA (*p* < 0.01) and phospho-Akt (*p* < 0.05) in PASMCs induced by hypoxia. These results suggest that ISL can attenuate MCT-induced PH via its anti-inflammatory and antiproliferative actions.

## 1. Introduction

Pulmonary hypertension (PH) is a progressive and severe disease characterized pathophysiologically by increased pulmonary pressure, pulmonary artery structural remodeling, and right ventricular hypertrophy and finally leads to death [1, 2]. Nowadays, there are three major classes of drugs, prostaglandins, phosphodiesterase inhibitors, and endothelin receptor antagonists available to treat PH, but they all present limited effectiveness and even produce unwanted consequences such as dysregulated pulmonary vasoconstriction response and worsened gas exchange [3, 4]. Therefore, there is an urgent need to develop new agents that are more effective and less adverse in treating PH. Fortunately, there are several types of animal PH models including monocrotaline (MCT)-induced rat PH, which pathophysiologically mimic human PH and help screen new chemical candidates for the treatment of PH. Studies have shown that a single dose of MCT can elicit the typical PH pathophysiologies in rat cardiopulmonary system, which share similar processes with those in human PH. For example, MCT administration engenders a persistent elevation of pulmonary vascular resistance and a progressive increase in the artery pressure followed by pulmonary artery structural remodeling and right ventricular hypertrophy consequently results in collapse of respiratory and circulatory systems [5]. Thus, impeding or reversing the pathophysiological progress of MCT-induced PH is a promising approach to treat chemicals-induced PH.

So far, the molecular mechanisms of PH are not fully understood, but clinical and preclinical evidences show inflammation is a key player in mediating the pathophysiological process of PH [6]. In PH patients, there are significantly increased circulating levels of proinflammatory cytokines such as interleukin-6 (IL-6) and tumor necrosis factor-*α* (TNF-*α*), and the high levels of these factors are associated with the increased risk of death in the patients [7]. Likewise, in animal models, the degree of perivascular inflammation positively correlates with pulmonary vascular remodeling [8]. IL-6 and TNF-*α* can induce the proliferation, migration, and differentiation of pulmonary vascular cells, harmfully promoting vascular remodeling [9]. In addition, proinflammatory factors directly disrupt endothelial membrane integrity and enhance hyperplasia in pulmonary artery smooth muscle cells (PASMCs), and the latter is another key player in the progress of PH [9]. It is turned out that the excessive PASMCs proliferation is mainly responsible for the pulmonary vascular medial hyperplasia, elevated vascular resistance, and right ventricular hypertrophy [1, 10]. Therefore, dampening the inflammatory response and inhibiting the abnormal proliferation of PASMCs can provide an effective way to halt the development of PH pathology.

*Glycyrrhizae* radix (*G.* radix, licorice) is most frequently used in traditional Chinese medicine to treat a variety of pathological states due to its superb detoxification effects [11]. Isoliquiritigenin (ISL) is an important bioactive ingredient in* G.* radix, mediates the therapeutic effects of* G.* radix, and possesses a wide range of pharmacological properties such as anti-inflammatory, antioxidant, antitumor, and immunoregulatory activities [12–16]. ISL inhibits carbon tetrachloride-induced hepatic damage and the proliferation of human prostate cancer LNCaP cells through its antioxidant and anti-inflammatory actions [17, 18]. ISL improves atherosclerosis and hypoxia-induced cardiomyocytes contractile dysfunction by decreasing the levels of reactive oxygen species (ROS) and proinflammatory factors [19–21]. These findings collectively indicate that ISL may have a therapeutic effect on PH. Indeed, in a recent study, we have found ISL alleviated hypoxic PH through inhibition of oxidative stress, indicating a potential application of ISL to treat PH. As mentioned above, PH presents common pathophysiologies in its final phase, but involves different etiologies that complicate its pharmacotherapies. Therefore, to support the therapeutic effect of ISL on hypoxic PH and further expand the therapeutic applications of ISL in treating PH, in the present study, we evaluated the effects of ISL on chemicals-induced PH in rats. For this, we first established an MCT-induced PH rat model where exacerbated inflammation and excessive proliferation of PASMCs play a key role and then by using this model examined the effect of ISL on rat MCT-induced PH and investigated the relevant mechanisms.

## 2. Materials and Methods

### 2.1. Reagents and Antibodies

ISL > 99% purity was provided by Shanghai Yuanye Biotechnology Co., Ltd. (Shanghai, China). Monocrotaline (MCT) was purchased from Sigma Sigma–Aldrich Inc. (St. Louis, MO, USA). Fetal bovine serum (FBS), Dulbecco's modified Eagle's medium (DMEM), Trizol reagent, and trypsin solution were obtained from Gibco Laboratories (Grand Island, NY, USA). Antibodies against *α*-smooth muscle actin (*α*-SMA), proliferating cell nuclear antigen (PCNA), Akt, phospho-Akt (p-Akt), and GAPDH were purchased from Cell Signaling Technology (Beverly, MA, USA).

### 2.2. Animals and Experimental Design

Eight-week-old male Sprague–Dawley rats (body weight 220–250 g) were obtained from the Animal Center of Qiqihar Medical University, China. The rats were caged (3 rats per cage) and supplied by filtered pathogen-free air, unlimited commercial rat chow, and water at a temperature between 22 and 24°C with 12 h light/dark cycles and relative humidity of 50%. All animal experiments were approved by the Animal Care and Use Committee of the Qiqihar Medical University and conducted in accordance with the National Institutes of Health guidelines concerning the care and use of laboratory animals. After 7 days of acclimatization period, the rats were randomly divided into 4 groups: saline-treated group (*n* = 8), MCT-treated control group (*n* = 8), MCT + 10 mg/kg/d ISL (MCT+ISL10) group (*n* = 8), and MCT + 30 mg/kg/d ISL (MCT+ISL30) group (*n* = 8). For the establishment of an MCT-induced PH rat model, rats were given a single intraperitoneal injection of MCT (50 mg/kg), which was dissolved in 1N hydrochloric acid, diluted in sterile saline, and adjusted to pH 7.4 with 1N sodium hydroxide. Thereafter, the rats stayed in their home cages for 28 days and received ISL treatments. ISL was dissolved in 5% Tween-80 and intragastrically administered to the rats at the dose of 10 mg/kg/d or 30 mg/kg/d once a day for 28 days, while an equal volume of 5% Tween-80 was given to the rats in the control groups. Thirty minutes after the final dose of ISL, the rats were subjected to hemodynamic analysis, and then blood samples and lungs of the rats were collected for further biochemical and morphologic assays.

### 2.3. Hemodynamic Assay and Assessment of the Right Ventricular Hypertrophy

The rats were anesthetized with an intraperitoneal injection of 7.5 mL/kg 4.8% tribromoethanol, and a polyethylene catheter was inserted into the right ventricle through the right jugular vein. The right ventricle systolic pressure (RVSP) was measured via a fluid-filled pressure transducer and recorded using PowerLab Software (ADInstruments, Castle Hill, Australia). For assessment of right ventricular hypertrophy, the left ventricular wall plus septum and the right ventricular wall were harvested, and the weight ratio of the right ventricular wall/left ventricular wall plus septum (Fulton index) was calculated to quantify the right ventricular hypertrophy.

### 2.4. Histological Observation

A lobe of the right lung from each rat was fixed in 4% neutral-buffered formalin, subjected to paraffin embedding, sliced into 4-*μ*m thick sections, and then stained with hematoxylin and eosin (HE). Morphologic changes in small pulmonary arteries (range 50–200 *μ*m) were observed. For assessment of pulmonary artery structural remodeling, the total vessel area, the medial wall area, the inside diameter, and outside diameter of pulmonary arteries were measured. Pulmonary artery structural remodeling was evaluated by the percentage medial wall area (WA%) = (medial wall area)/(total vessel area) × 100 and the percentage medial wall thickness (WT%) = (outside diameter−inside diameter)/(outside diameter)×100.

### 2.5. Immunohistochemical Staining

Lung sections were deparaffinized in xylene and rehydrated with ethanol, and antigens were retrieved. After blocking unspecific protein binding with 5% bovine serum albumin for 30 min at room temperature, the lung sections were incubated overnight at 4°C with one of the following antibodies: anti-PCNA antibody (1:1500) or anti-*α*-SMA antibody (1:500). Then, sections were further incubated with a biotinylated anti-mouse IgG antibody for 2 h at room temperature. Finally, the sections were visualized using diaminobenzidine and counterstained with hematoxylin. Quantitative assessments of *α*-SMA and PCNA were carried out as described previously [22].

### 2.6. Assay of IL-6 and TNF-*α*

The blood samples were centrifuged at 1,500 x g, at 4°C for 20 minutes, and the sera were collected and the serum levels of IL-6 and TNF-*α* were measured using enzyme-linked immunosorbent assay (ELISA) kits (Shanghai Bluegene Biotech Co., Ltd., Shanghai, China) according to the manufacturer's instructions.

### 2.7. Quantitative Real-Time RT-PCR Analysis

Total RNA was extracted from lung tissues using Trizol agent. Quantitative real-time RT-PCR (qPCR) was performed to assess mRNA expression of the following genes. The primers for the genes were as follows: IL-6 (101 bp) forward: 5′-AAGTCGGAGGCTTAATTACACATGT-3′, reverse: 5′-AAGTGCATCATCGTTGTTCATACA-3′; TNF-*α* (110 bp): forward: 5′-AAATGGGCTCCCTCTATCAGTTC-3′, reverse: 5′-TCTGCTTGGTGGTTTGCTACGAC-3′; housekeeping gene GAPDH (123 bp): forward: 5′-AGGTCGGTGTGAACGGATTTG-3′, reverse: 5′- TGTAGACCATGTAGTTGAGGTCA -3′, respectively.

### 2.8. Primary PASMCs Culture and In Vitro Hypoxia

Rat primary PASMCs were obtained by explants method and cultured as described previously [22]. Smooth muscle cell identity was verified by *α*-SMA immunocytochemical staining, and the cells of passages 3–7 were used. PASMCs were divided into 6 groups: normoxia, hypoxia, hypoxia + 10 *μ*M ISL, hypoxia + 30 *μ*M ISL, and hypoxia + 100 *μ*M ISL. The hypoxia or normoxia was implemented by exposing PASMCs to either in 3% oxygen or 21% oxygen condition for 24 h, respectively.

### 2.9. Cell Proliferation Assay

PASMCs proliferation was measured by the 3-(4,5-dimethylthiazol-2-yl)-2, 5-diphenyltetrazolium bromide (MTT) assay. PASMCs were seeded in 96-well culture plates (2×10^4^ cells per well) and incubated for 24 h under the condition of either normoxia or hypoxia plus ISL, and then 10 *μ*L MTT (5 mg/mL) was added to each well. After being incubated with MTT for 4 h, the media of each well was removed, and the produced formazan crystals in the wells were dissolved by adding 150 *μ*L dimethyl sulfoxide (DMSO). The optical density value of each well was read at 490 nm wavelength using a spectrophotometer, which is directly proportional to the number of living cells.

### 2.10. Western Blotting Analysis

PASMCs were lysed in a protein extraction buffer. A cocktail of protease and phosphatase inhibitors was added to the buffer in advance. Then the lysates were centrifuged at 12,000 rpm for 15 min at 4°C, and the supernatants were collected. The protein concentration was determined by using the Lowry method (Bio-Rad). Twenty-five *μ*g of protein was separated by electrophoresis on 10% sodium dodecyl sulfate polyacrylamide gels and transferred to a nitrocellulose membrane. The membrane was blocked and incubated with the appropriate primary antibody to PCNA (1:1000), p-Akt (1:1000), and total Akt (1:1000). Immunoreactive proteins were detected by chemiluminescence with an ECL detection system.

### 2.11. Statistical Analyses

All values are expressed as means ± SEM. Statistical analysis was performed using the commercially available software GraphPad Prism 5.0 (GraphPad Software, San Diego, CA, USA). The statistical significance of differences between groups was evaluated by one-way analysis of variance (ANOVA) with Newman-Keuls multiple-comparison tests. Significant difference was accepted at* p *< 0.05.

## 3. Results

### 3.1. ISL Attenuated MCT-Induced PH and Pulmonary Artery Remodeling

**Figure 1:**
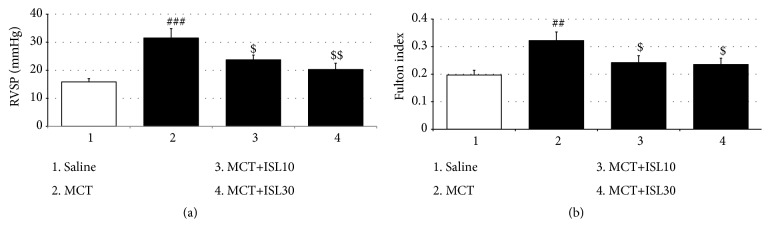
Effects of ISL on pulmonary hypertension and right ventricular hypertrophy in MCT-induced PH. (a) Right ventricular systolic pressure (RVSP) in rats. (b) Fulton index in rats. Values are means ± SEM (*n* = 8). ^##^* p *< 0.01; ^###^* p* < 0.001, compared with saline group; ^$^* p *< 0.05; ^$$^* p* < 0.01, compared with MCT group.

As seen in Figure 1(a), a single MCT caused a substantial PH as evidenced by significant increases in the average RVSP of the MCT-treated control rats [F_(3, 28)_ = 8.59,* p* < 0.001, saline group vs. MCT group,* p* < 0.001]. However, both doses of ISL (10 mg/kg/d and 30 mg/kg/d) prevented these increases (MCT group vs. MCT+ISL10,* p* < 0.05; MCT group vs. MCT+ISL30,* p* < 0.01) (Figure 1(a)). In agreement with the RVSP results, MCT induced a significant elevation of the Fulton indices [F_(3, 28)_ = 4.59,* p* < 0.01, saline group vs. MCT group,* p* < 0.01], which was also blocked by both doses of ISL treatment (MCT group vs. MCT+ISL10,* p* < 0.05; MCT group vs. MCT+ISL30,* p* < 0.05) (Figure 1(b)).

**Figure 2:**
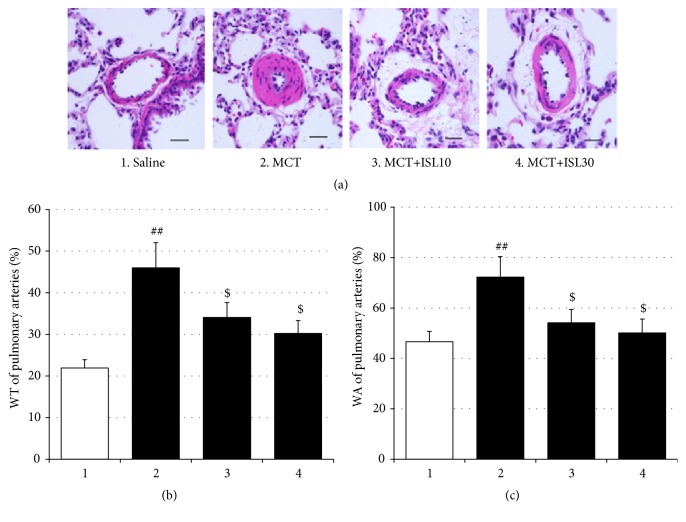
Effects of ISL on pulmonary artery structural remodeling. (a) Hematoxylin and eosin staining of pulmonary arteries (scale bar = 50 *μ*m). (b) Medial wall thickness (WT%) of pulmonary arteries. (c) Medial wall area (WA%) of pulmonary arteries. Values are means ± SEM (*n* = 8). ^##^* p* < 0.01, compared with saline group; ^$^* p* < 0.05, compared with MCT group.

In the present study, MCT-induced pulmonary artery remodeling was indexed by WT% and WA% of pulmonary arteries. As shown in Figure 2, MCT markedly elevated WT% and WA% [WT%: F_(3, 28)_ = 6.41,* p* < 0.01; saline group vs. MCT group,* p* < 0.01; WA%: F_(3, 28)_ = 4.51,* p* < 0.05; saline group vs. MCT group,* p* < 0.01], while treatment with ISL improved these pathological changes (WT%: MCT group vs. MCT+ISL10,* p* < 0.05; MCT group vs. MCT+ISL30,* p* < 0.05; WA%: MCT group vs. MCT+ISL10,* p* < 0.05; MCT group vs. MCT+ISL30,* p* < 0.05) (Figure 2).

### 3.2. ISL Blocked an Elevation of *α*-SMA Expressions

**Figure 3:**
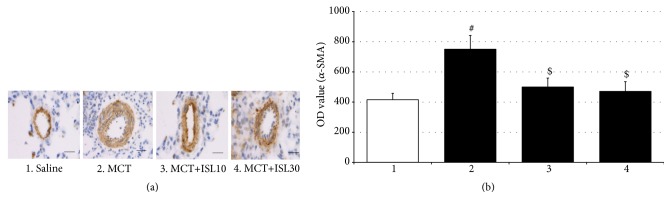
Analysis of optical density (OD) value of *α*-SMA in pulmonary arteries. (a) Immunohistochemical staining of *α*-SMA of pulmonary arteries (scale bar = 50 *μ*m). (b) Quantitative analysis of OD value of *α*-SMA immunoreactivity in pulmonary arteries. Values are means ± SEM (*n* = 5). ^#^* p* < 0.05, compared with saline group; ^$^* p* < 0.05, compared with MCT group.

The hyperplastic smooth muscularization was examined in lung sections using an antibody against *α*-SMA to evaluate the cellular mechanism for the increased thickness and area of pulmonary arteries. As seen in Figure 3, the integrated optical density (OD) value of *α*-SMA in the MCT control group was higher than in the saline control group [F_(3, 16)_ = 4.93,* p* < 0.05, saline group vs. MCT group,* p* < 0.05], reflecting the association of increased thickness and area of pulmonary arteries with enhanced proliferation of SMCs. However, in accordance with the abovementioned morphological observation, both doses of ISL blocked the MCT-induced enhancement of the OD value of *α*-SMA (MCT group vs. MCT+ISL10,* p* < 0.05; MCT group vs. MCT+ISL30,* p* < 0.05) (Figure 3).

### 3.3. ISL Reduced MCT-Induced Plasma IL-6 and TNF-*α* Secretion

**Figure 4:**
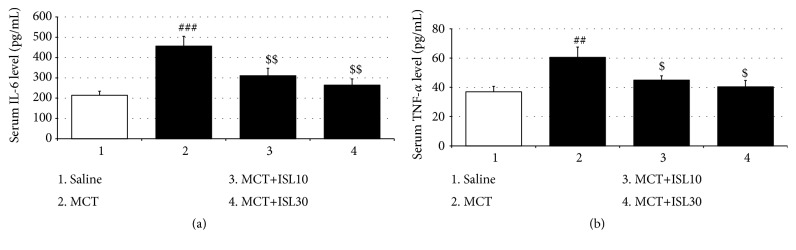
Effects of ISL on the levels of IL-6 and TNF-*α* in serum. (a) IL-6 levels in serum. (b) TNF-*α* levels in serum. Values are means ± SEM (*n* = 8). ^##^* p* < 0.01; ^###^* p* < 0.001, compared with saline group; ^$^* p* < 0.05; ^$$^* p* < 0.01, compared with MCT group.

The ELISA analysis revealed that the serum levels of both IL-6 and TNF-*α* in the MCT control group were significantly higher than those in the saline control group [IL-6: F_(3, 28)_ = 8.66,* p* < 0.001, saline group vs. MCT group,* p* < 0.001; TNF-*α*: F_(3, 28)_ = 4.79,* p* < 0.01, saline group vs. MCT group,* p* < 0.01]. However, these increases in the inflammatory factor levels were reduced by treatment with both doses of ISL (IL-6: MCT group vs. MCT+ISL10,* p* < 0.01; MCT group vs. MCT+ISL30,* p* < 0.01; TNF-*α*: MCT group vs. MCT+ISL10,* p* < 0.05; MCT group vs. MCT+ISL30,* p* < 0.05) (Figure 4).

### 3.4. ISL Prevented MCT-Induced Upregulation of Pulmonary Expressions of IL-6 and TNF-*α* mRNA

**Figure 5:**
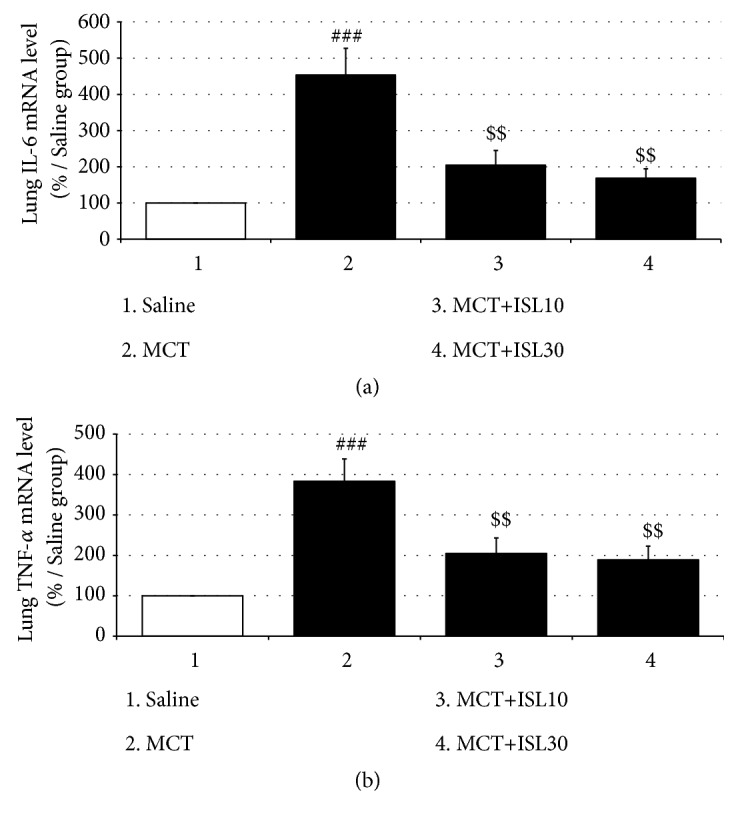
Effects of ISL on IL-6 and TNF-*α* mRNA expressions in rat lungs. (a) IL-6 mRNA levels in rat lungs. (b) TNF-*α* mRNA levels in rat lungs. Values are means ± SEM (*n* = 4). ^###^* p* < 0.001, compared with saline group; ^$$^* p* < 0.01, compared with MCT group.

Being paralleled with the plasma ELISA results, the qPCR analysis showed that a single MCT increased the mRNA levels of IL-6 and TNF-*α* in lung tissues [IL-6 mRNA: F_(3, 12)_ = 12.17,* p* < 0.001, saline group vs. MCT group,* p* < 0.001; TNF-*α* mRNA: F_(3, 12)_ = 10.05,* p* < 0.01, saline group vs. MCT group,* p* < 0.001]. However, the same qPCR assay also revealed both doses of ISL prevented this upregulation of the mRNA expressions (IL-6 mRNA: MCT group vs. MCT+ISL10,* p* < 0.01; MCT group vs. MCT+ISL30,* p* < 0.01; TNF-*α* mRNA: MCT group vs. MCT+ISL10,* p* < 0.01; MCT group vs. MCT+ISL30,* p* < 0.01) (Figure 5).

### 3.5. ISL Inhibited PASMCs Proliferation

**Figure 6:**
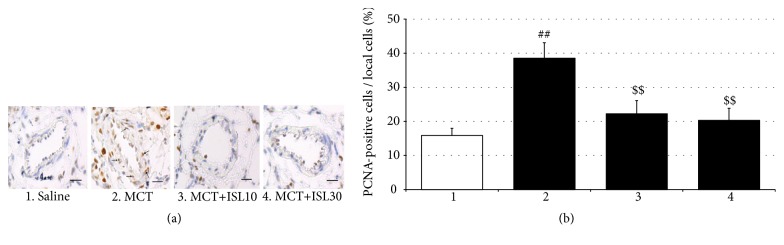
Analysis of the percentage of PCNA-positive cells in the medial wall of pulmonary arteries. (a) Immunohistochemical staining of PCNA of pulmonary arteries (scale bar = 50 *μ*m). (b) The percentage of the PCNA-positive cells in the total smooth muscle cells in the medial wall of pulmonary arteries. Values are means ± SEM (*n* = 5). ^##^* p* < 0.01, compared with saline group; ^$$^* p* < 0.01, compared with MCT group.

PCNA is a marker for cell proliferation. To determine the effect of ISL on PASMCs proliferation, the PCNA-positive cells in the medial wall of pulmonary arteries were quantified via the immunohistochemistry staining. There were increased numbers of PCNA-positive cells in pulmonary arteries from the MCT-challenged rats compared to the saline-treated control rats [F_(3, 16)_ = 7.12,* p* < 0.01, saline group vs. MCT group,* p* < 0.01], which was inhibited by treatment with ISL (MCT group vs. MCT+ISL10,* p* < 0.01; MCT group vs. MCT+ISL30,* p* < 0.01) (Figure 6).

**Figure 7:**
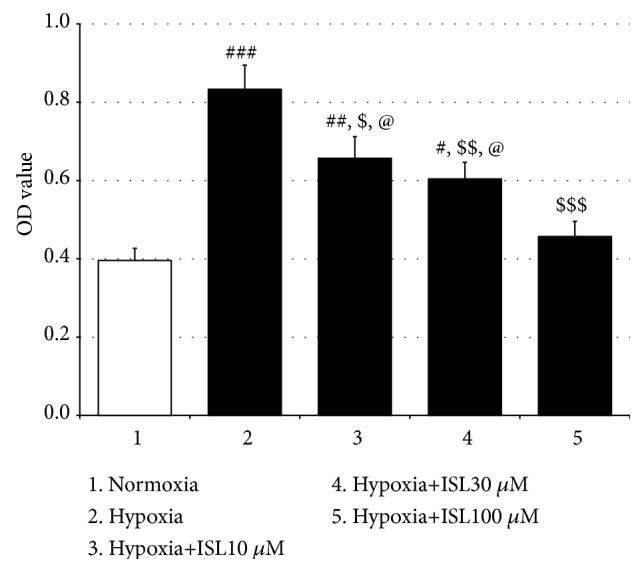
Effects of ISL on hypoxia-induced PASMCs proliferation. Values are means ± SEM (*n* = 5). ^#^* p* < 0.05, ^##^* p* < 0.01, and ^###^* p* < 0.001, compared with normoxia group; ^$^* p* < 0.05, ^$$^* p* < 0.01, and ^$$$^* p* < 0.001, compared with hypoxia group; ^@^* p* < 0.05, compared with Hypoxia+ISL 100 *μ*M group.

**Figure 8:**
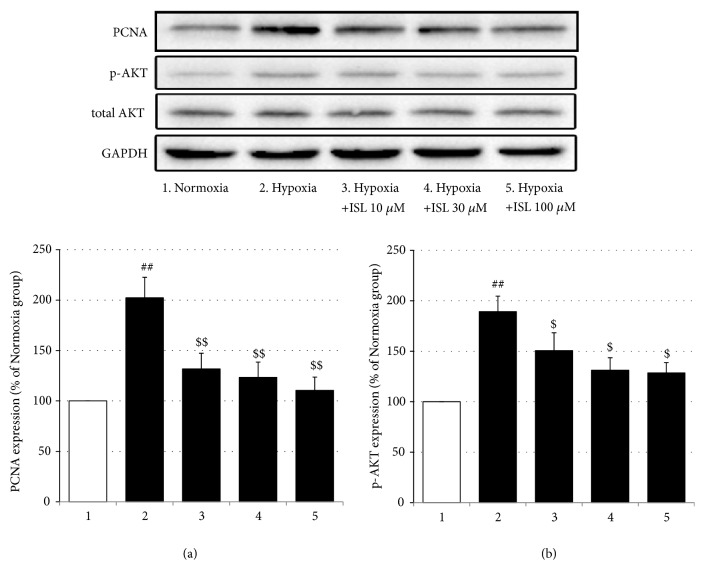
Effects of ISL on hypoxia-induced protein expressions of PCNA and p-Akt in PASMCs. (a) PCNA protein expressions (relative to normoxia group). (b) p-Akt protein expressions (relative to normoxia group). Values are means ± SEM (*n* = 4). ^##^* p* < 0.01, compared with normoxia group; ^$^* p* < 0.05 and ^$$^* p* < 0.01, compared with hypoxia group.

In addition, in the* in vitro* experiments, the proliferation of the PASMCs under the hypoxia condition was greater than that under the normoxia condition [F_(4, 20)_ = 13.29,* p* < 0.001, normoxia group vs. hypoxia group,* p* < 0.001], but treatment with all three doses of ISL inhibited this increased proliferation in a dose-dependent way (Hypoxia vs. Hypoxia+ISL 10 *μ*M,* p* < 0.05; Hypoxia vs. Hypoxia+ISL 30 *μ*M,* p* < 0.01, Hypoxia vs. Hypoxia+ISL 100 *μ*M,* p* < 0.001; Hypoxia+ISL 10 *μ*M vs. Hypoxia+ISL 100 *μ*M,* p* < 0.05; Hypoxia+ISL 30 *μ*M vs. Hypoxia+ISL 100 *μ*M,* p* < 0.05) (Figure 7). Moreover, in Western blotting analysis, hypoxia elevated the PCNA and p-Akt protein expressions in the cultured PASMCs [PCNA: F_(4, 15)_ = 7.60,* p* < 0.01, Normoxia vs. Hypoxia,* p* < 0.01; p-Akt: F_(4, 15)_ = 6.79,* p* < 0.01, normoxia group vs. hypoxia group,* p* < 0.01] but did not significantly affect the total Akt expressions, while again, ISL treatment suppressed these increments (PCNA: Hypoxia vs. Hypoxia+ISL 10 *μ*M,* p* < 0.01; Hypoxia vs. Hypoxia+ISL 30 *μ*M,* p* < 0.01, Hypoxia vs. Hypoxia+ISL 100 *μ*M,* p* < 0.01; p-Akt: Hypoxia vs. Hypoxia+ISL 10 *μ*M,* p* < 0.05; Hypoxia vs. Hypoxia+ISL 30 *μ*M,* p* < 0.05; Hypoxia vs. Hypoxia+ISL 100 *μ*M,* p* < 0.05) (Figure 8).

## 4. Discussion

In this study, when tested at 28 days after a single dose of MCT, there were significant increases in pulmonary artery pressure and structural remodeling of pulmonary arteries along with exacerbated right ventricular hypertrophy in the MCT-challenged rats, marking the successful establishment of MCT-induced PH. However, in this study, oral treatment with both doses of ISL (10 mg/kg/d and 30 mg/kg/d) prevented these pathophysiological changes. In addition, ISL inhibited MCT-induced increases in serum levels and pulmonary gene expressions of IL-6 and TNF-*α*. Moreover, in the* in vitro* experiment, ISL dose-dependently inhibited hypoxia-induced increases of PASMCs proliferation, and suppressed hypoxia-induced PCNA and p-Akt protein expressions. These results collectively suggest that ISL produces therapeutic effects on MCT-induced PH, and that the effects are mediated through its anti-inflammatory and antiproliferative actions.

In the present study, we have found both doses of ISL reduced MCT-induced elevation of the RVSP, which is consistent with the previous founding that ISL attenuated chronic hypoxia-induced pulmonary hypertension [23]. This effect appears to be very important, because the increased pulmonary artery pressure is not only a typical resulting pathophysiology of PH but also an important causative factor for PH development. Increased pulmonary artery pressure evokes adjustment mechanisms in the body, which gradually lead to pathological changes in the cardiopulmonary system. Evidence has shown that ISL directly relaxes rat aorta smooth muscle and guinea-pig tracheal smooth muscle [24, 25], therefore, the inhibitory effect of ISL on the RVSP may be an initial mechanism that underlies the effects of ISL on the structural remodeling of pulmonary arteries and right ventricular hypertrophy in the MCT-induced PH rats.

Right ventricular hypertrophy is universally measured by the Fulton index. In the present study, a single dose of MCT resulted in a significant increase in the Fulton indices, indicating the occurrence of right ventricular hypertrophy; however, this was reversed by treatment with both doses of ISL. Moreover, in the present study, MCT administration induced pulmonary artery remodeling manifested by the significant increases in the WT% and WA% of pulmonary arteries, which were also inhibited by both doses of ISL. These effects conjunction with the effect of ISL on the RVSP suggest that ISL can attenuate all the three major pathophysiologies of PH induced by MCT in rats.

An excessively increased inflammatory state induced by adverse stimuli such as hypoxia and MCT is critical in the initiation and development of the pathophysiological changes in PH, particularly in MCT-induced PH rats [26]. Dulce* et al.* have reported MCT administration significantly increased the plasma IL-6 and TNF-*α* levels in rats [27]; Tiago* et al.* have found MCT markedly elevated the IL-6 mRNA expressions in lung tissues, which was associated with exacerbated hemodynamic and pathological changes in rat cardiopulmonary system [28]. In agreement with these reports, in the present study, the ELISA revealed the MCT enhanced serum levels of IL-6 and TNF-*α*, and the qPCR analysis found the MCT promoted pulmonary gene expressions of these two proinflammatory factors. Modulation of IL-6 and TNF-*α* can dampen or facilitate the process of PH. For example, immunosuppressive steroids decrease IL-6 levels and reduce pulmonary artery pressures [29]; high levels of TNF-*α* suppress the mRNA expression of the vasodilating prostacyclin and increase pulmonary vascular reactivity [30, 31], and in the aforementioned studies done by Dulce* et al.* and Tiago* et al.*, the pulmonary hemodynamics and the right ventricular hypertrophy in PH rats were ameliorated by antagonizing the overactivated IL-6 and TNF-*α* systems. Similarly, in the present study, treatment with ISL blocked MCT-induced augmentation of IL-6 and TNF-*α* expressions in the general circulation and lung tissues. It has been reported that ISL inhibits lipopolysaccharide-induced IL-6 and TNF-*α* production in bone marrow-derived dendritic cells [32] and suppressed carbon tetrachloride-induced hepatic TNF-*α* and cyclooxygenase-2 expressions [17]. Therefore, our results suggest that ISL can inhibit the inflammatory response in MCT-induced PH rats, by which ISL improves the pathophysiologies of the PH.

The pulmonary vascular wall consists of the fibroblast cells (adventitia), smooth muscle cells (media), and endothelial cells (intima). Of these 3 cell types, abnormal proliferation of medial smooth muscle cells is the main determinant of pulmonary vascular resistance and considered as a hallmark of pulmonary artery structural remodeling [33]. Systemically and locally produced inflammatory cytokines recruit a variety of intracellular signaling pathways including protein kinase C, mitogen-activated protein kinases, phosphatidylinositol-3 kinases (PI3K), and Ca^2+^/calmodulin-dependent protein kinases to control PASMCs contractility, differentiation, and proliferation [6, 9]. PCNA, an acidic nuclear protein, is expressed during the G1/S phase in the cell cycle and used as an operational marker for cell proliferation. Activation of PI3K/Akt induces proproliferative and antiapoptotic responses in PASMCs [34]. In the present study, the immunofluorescence staining showed that the MCT significantly increased the numbers of PCNA-positive cells in the medial wall of pulmonary arteries, and the MTT analysis revealed 24-h hypoxia notably elevated the proliferation of the primary cultured PASMCs. Furthermore, in the Western blotting analysis, the hypoxia enhanced the PCNA and p-Akt protein expressions. However, all these changes were inhibited by treatment with ISL in the present study. Evidence has shown that ISL inhibits the proliferation of some cancerous and noncancerous cells including human arterial smooth muscle cells [11, 35], and this effect is mediated through the PI3K/Akt signaling pathway [36]. Therefore, these results indicate that ISL can inhibit hypoxia-induced proliferation of PASMCs via modulation of the PI3K/Akt pathway, which may be linked to the therapeutic effect of ISL on MCT-induced PH.

It is worth noting that the doses of ISL used in the in vivo or in the in vitro experiments in the present study effectively improved pathophysiological and biochemical changes related to PH, but an expected dose-dependent fashion was not statistically observed in the checked effects except for the effect on the proliferation of the PASMCs. This failure to observe a dose-dependent response in the present study may be mainly ascribed to the inadequate dose escalation. Considering the importance of the dose-response relationship in the pharmacodynamics of a drug, this limitation must be improved in the future work.

In summary, in the present study, 10 and 30 mg/kg/d ISL attenuated the pathophysiological changes in the hemodynamics, pulmonary artery structural remodeling, and right ventricle hypertrophy induced by MCT administration in rats. In addition, ISL inhibited MCT-induced inflammatory response, reversed abnormal PASMCs proliferation in* in vivo* and* in vitro, * and moreover, blocked hypoxia-induced Akt phosphorylation* in vitro*. These observations suggest that ISL exerts therapeutic effects on MCT-induced PH via inhibition of the inflammatory response and PASMCs proliferation and further provide an experimental basis for expanding the therapeutic applications of ISL to treatment of chemicals-induced PH.

## Data Availability

The data supporting the conclusions of the present study are properly analyzed and included in Results section and are available from the corresponding author upon reasonable request.

## References

[1] Hoeper M. M., Ghofrani H., Grünig E., Klose H., Olschewski H., Rosenkranz S. (2017). Pulmonary hypertension. *Deutsches Arzteblatt International*.

[2] Bazan I. S., Fares W. H. (2015). Pulmonary hypertension: diagnostic and therapeutic challenges. *Therapeutics and Clinical Risk Management*.

[3] Zamanian R. T., Kudelko K. T., Sung Y. K., de Jesus Perez V., Liu J., Spiekerkoetter E. (2014). Current clinical management of pulmonary arterial hypertension. *Circulation Research*.

[4] Hambly N., Alawf F., Mehta S. (2016). Pulmonary hypertension: diagnostic approach and optimal management. *Canadian Medical Association Journal*.

[5] Hussain A., Suleiman M. S., George S. J., Loubani M., Morice A. (2017). Hypoxic pulmonary vasoconstriction in humans: tale or myth. *The Open Cardiovascular Medicine Journal*.

[6] Pugliese S. C., Poth J. M., Fini M. A., Olschewski A., El Kasmi K. C., Stenmark K. R. (2015). The role of inflammation in hypoxic pulmonary hypertension: from cellular mechanisms to clinical phenotypes. *American Journal of Physiology-Lung Cellular and Molecular Physiology*.

[7] Soon E., Holmes A. M., Treacy C. M. (2010). Elevated levels of inflammatory cytokines predict survival in idiopathic and familial pulmonary arterial hypertension. *Circulation*.

[8] Groth A., Vrugt B., Brock M., Speich R., Ulrich S., Huber L. C. (2014). Inflammatory cytokines in pulmonary hypertension. *Respiratory Research*.

[9] Rabinovitch M., Guignabert C., Humbert M., Nicolls M. R. (2014). Inflammation and immunity in the pathogenesis of pulmonary arterial hypertension. *Circulation Research*.

[10] Malenfant S., Neyron A.-S., Paulin R. (2013). Signal transduction in the development of pulmonary arterial hypertension. *Pulmonary Circulation*.

[11] Peng F., Du Q., Peng C. (2015). A review: the pharmacology of isoliquiritigenin. *Phytotherapy Research*.

[12] Gaur R., Kumar S., Trivedi P. (2010). Liquiritigenin derivatives and their hepatotoprotective activity. *Natural Product Communications (NPC)*.

[13] Kim S. C., Park S. J., Lee J. R., Seo J. C., Yang C. H., Byun S. H. (2008). Cytoprotective activity of Glycyrrhizae radix extract against arsenite-induced cytotoxicity. *Evidence-Based Complementary and Alternative Medicine*.

[14] Yang E. J., Min J. S., Ku H. Y. (2012). Isoliquiritigenin isolated from Glycyrrhiza uralensis protects neuronal cells against glutamate-induced mitochondrial dysfunction. *Biochemical and Biophysical Research Communications*.

[15] Lee S. H., Kim J. Y., Seo G. S., Kim Y.-C., Sohn D. H. (2009). Isoliquiritigenin, from *Dalbergia odorifera*, up-regulates anti-inflammatory heme oxygenase-1 expression in RAW264.7 macrophages. *Inflammation Research*.

[16] Kwon G. T., Cho H. J., Chung W., Park K., Moon A., Park J. H. (2009). Isoliquiritigenin inhibits migration and invasion of prostate cancer cells: possible mediation by decreased JNK/AP-1 signaling. *The Journal of Nutritional Biochemistry*.

[17] Zhao Z., Park S. M., Guan L. (2015). Isoliquiritigenin attenuates oxidative hepatic damage induced by carbon tetrachloride with or without buthionine sulfoximine. *Chemico-Biological Interactions*.

[18] Zhang X., Yeung E. D., Wang J. (2010). Isoliquiritigenin, a natural anti-oxidant, selectively inhibits the proliferation of prostate cancer cells. *Clinical Experimental Pharmacollogy & Physiology*.

[19] Zhang X., Zhu P., Zhang X. (2013). Natural antioxidant-isoliquiritigenin ameliorates contractile dysfunction of hypoxic cardiomyocytes via AMPK signaling pathway. *Mediators of Inflammation*.

[20] Du F., Gesang Q., Cao J. (2016). Isoliquiritigenin attenuates atherogenesis in apolipoprotein E-deficient mice. *International Journal of Molecular Sciences*.

[21] Noguchi C., Yang J., Sakamoto K. (2008). Inhibitory effects of isoliquiritigenin and licorice extract on voltage-dependent K+ currents in H9c2 cells. *Journal of Pharmacological Sciences*.

[22] Jin H., Liu M., Zhang X. (2016). Grape seed procyanidin extract attenuates hypoxic pulmonary hypertension by inhibiting oxidative stress and pulmonary arterial smooth muscle cells proliferation. *The Journal of Nutritional Biochemistry*.

[23] Zhang S., Li X., Yao L. (2018). Effects of isoliquiritigenin on pulmonary vascular remodeling in chronic hypoxia rat model. *Acta Anatomica Sinica*.

[24] Yu S., Kuo S. (1995). Vasorelaxant effect of isoliquiritigenin, a novel soluble guanylate cyclase activator, in rat aorta. *British Journal of Pharmacology*.

[25] Liu B., Yang J., Wen Q., Li Y. (2008). Isoliquiritigenin, a flavonoid from licorice, relaxes guinea-pig tracheal smooth muscle in vitro and in vivo: role of cGMP/PKG pathway. *European Journal of Pharmacology*.

[26] Nogueira-Ferreira R., Vitorino R., Ferreira R., Henriques-Coelho T. (2015). Exploring the monocrotaline animal model for the study of pulmonary arterial hypertension: a network approach. *Pulmonary Pharmacology and Therapeutics*.

[27] Fontoura D., Oliveira‐Pinto J., Tavares‐Silva M. (2014). Myocardial and anti‐inflammatory effects of chronic bosentan therapy in monocrotaline‐induced pulmonary hypertension. *Revista Portuguesa de Cardiologia (English Edition)*.

[28] Henriques-Coelho T., Oliveira S. M., Moura R. S. (2008). Thymulin inhibits monocrotaline-induced pulmonary hypertension modulating interleukin-6 expression and suppressing p38 pathway. *Endocrinology*.

[29] Bhargava A., Kumar A., Yuan N., Gewitz M. H., Mathew R. (1999). Monocrotaline induces interleukin-6 mRNA expression in rat lungs. *Heart Disease*.

[30] Itoh A., Nishihira J., Makita H., Miyamoto K., Yamaguchi E., Nishimura M. (2003). Effects of IL-1*β*, TNF-*α* and macrophage migration inhibitory factor on prostacyclin synthesis in rat pulmonary artery smooth muscle cells. *Respirology*.

[31] Fujita M., Shannon J. M., Irvin C. G. (2001). Overexpression of tumor necrosis factor-*α* produces an increase in lung volumes and pulmonary hypertension. *American Journal of Physiology-Lung Cellular and Molecular Physiology*.

[32] Li W., Sun Y. N., Yan X. T. (2014). Flavonoids from Astragalus membranaceus and their inhibitory effects on LPS-stimulated pro-inflammatory cytokine production in bone marrow-derived dendritic cells. *Archives of Pharmacal Research*.

[33] Shimoda L. A., Laurie S. S. (2013). Vascular remodeling in pulmonary hypertension. *Journal of Molecular Medicine*.

[34] Garat C. V., Crossno J. T., Sullivan T. M., Reusch J. E. B., Klemm D. J. (2013). Inhibition of phosphatidylinositol 3-kinase/akt signaling attenuates hypoxia-induced pulmonary artery remodeling and suppresses CREB depletion in arterial smooth muscle cells. *Journal of Cardiovascular Pharmacology*.

[35] Chen T., Deng S., Lin R. (2017). The inhibitory effect of Isoliquiritigenin on the proliferation of human arterial smooth muscle cell. *BMC Pharmacology & Toxicology*.

[36] Chen J., Liu C., Yang Q.-Q. (2018). Isoliquiritigenin suppresses osteosarcoma U2OS Cell proliferation and invasion by regulating the PI3K/Akt signalling pathway. *Chemotherapy*.

